# Correction to: Burosumab in X-linked hypophosphatemia: a profile of its use in the USA

**DOI:** 10.1007/s40267-018-0577-0

**Published:** 2018-11-12

**Authors:** Katherine A. Lyseng-Williamson

**Affiliations:** Springer, Private Bag 65901, Mairangi Bay, 0754 Auckland, New Zealand

## Correction to: Drugs & Therapy Perspectives (2018) 34(14):497–506 10.1007/s40267-018-0560-9

The article Burosumab in X-linked hypophosphatemia: a profile of its use in the USA, written by Katherine A. Lyseng-Williamson, was originally published Online First without open access. After publication in volume 34, issue 11, pages 497–506, Ultragenyx Pharmaceutical Inc. in partnership with Kyowa Kirin International plc requested that the article be Open Choice to make the article an open access publication. Post-publication open access was funded by Ultragenyx Pharmaceutical Inc. in partnership with Kyowa Kirin International plc. The article is forthwith distributed under the terms of the Creative Com_mons Attribution-NonCommercial 4.0 International License (http://creativecommons.org/licenses/by-nc/4.0/), which permits any noncommercial use, duplication, adaptation, distribution and reproduction in any medium or format, as long as you give appropriate credit to the original author(s) and the source, provide a link to the Creative Commons license and indicate if changes were made.

The original article has been updated.

